# Development of a Model to Estimate the Optimal Number of Oocytes to Attempt to Fertilize During Assisted Reproductive Technology Treatment

**DOI:** 10.1001/jamanetworkopen.2022.49395

**Published:** 2023-01-03

**Authors:** Katharine F. B. Correia, Stacey A. Missmer, Rachel Weinerman, Elizabeth S. Ginsburg, Brooke V. Rossi

**Affiliations:** 1 Department of Mathematics and Statistics, Amherst College, Amherst, Massachusetts; 2 Department of Epidemiology, Harvard T.H. Chan School of Public Health, Boston, Massachusetts; 3 Department of Obstetrics, Gynecology, and Reproductive Biology, College of Human Medicine, Michigan State University, Grand Rapids; 4 Division of Reproductive Endocrinology and Infertility, University Hospitals Cleveland Medical Center, Beachwood, Ohio; 5 Center for Infertility and Reproductive Surgery, Department of Obstetrics, Gynecology, and Reproductive Biology, Brigham and Women’s Hospital and Harvard Medical School, Boston, Massachusetts; 6 Ohio Reproductive Medicine, Columbus

## Abstract

**Importance:**

Surplus cryopreserved embryos pose a challenge for in vitro fertilization patients and clinics; with Roe v. Wade overturned, some states may deem the discarding of surplus embryos illegal, radically changing in vitro fertilization practice. An evidence-based tool would help limit surplus embryo creation.

**Objective:**

To develop a prediction tool for determining how many oocytes should be exposed to sperm to create embryos to conserve the chance of live birth while minimizing surplus embryos.

**Design, Setting, and Participants:**

This diagnostic study used data from member clinics of the Society for Assisted Reproductive Technology Clinical Outcomes Reporting System between 2014 to 2019. A total of 410 719 oocyte retrievals and 460 577 embryo transfer cycles from 311 237 patients aged 18 to 45 years old who initiated their first oocyte stimulation cycle between January 1, 2014, and December 31, 2019, were included. Data were analyzed from February to June 2022.

**Exposures:**

Female patient age, anti-mullerian hormone level, diminished ovarian reserve diagnosis, number of oocytes retrieved, and the state where the clinic is located were included in the final models.

**Main Outcomes and Measures:**

The algorithm was based on 3 models with outcomes: (1) day of transfer; (2) proportion of retrieved oocytes that become usable blastocysts; and (3) number of blastocysts needed for transfer for 1 live birth to occur.

**Results:**

The median (IQR) age at stimulation cycle start was 35 (29-32) years and the median (IQR) number of oocytes retrieved was 10 (6-17). The likelihood of recommending that all oocytes be exposed to sperm increased with age; less than 20.0% of retrievals among patients younger than 32 years and more than 99.0% of retrievals among patients older than 42 years received recommendations that all oocytes be exposed to sperm. Among cycles recommended to expose fewer than all oocytes, the median (IQR) numbers recommended for 1 live birth were 7 oocytes (7-8) for patients aged less than 32 years, 8 (7-8) for patients aged 32 to 34 years, and 9 (9-11) for patients aged 35 to 37 years.

**Conclusions and Relevance:**

In this diagnostic study of in vitro fertilization cycles, a prediction tool was developed to aid clinicians in determining the optimal number of oocytes to expose to sperm, reducing the number of unused embryos created and immediately addressing current patient and clinician concerns.

## Key Points

**Question:**

How many oocytes should be exposed to sperm during an assisted reproductive technology cycle to minimize the number of unused embryos while optimizing the probability of a live birth?

**Findings:**

In this national registry–based diagnostic study, in 66.2% of cycles among patients younger than 38 years, fewer than all retrieved oocytes could be exposed to sperm to minimize the number of unused embryos while optimizing the probability of a live birth.

**Meaning:**

These findings suggest the developed prediction tool could reduce the number of unused embryos created and immediately address current patient and clinician concerns.

## Teaser

This diagnostic study estimates the optimal number of oocytes to attempt to fertilize during assisted reproductive technology treatment.

## Introduction

The number of assisted reproductive technology (ART) cycles conducted each year continues to climb, with more than 2.5 million cycles performed worldwide in 2019.^1^ As the effectiveness of ART has greatly improved over the last few decades, more high-quality embryos are being created in each cycle, and a live birth is achievable with fewer embryos. The percentage of single embryo transfers in the US increased from 18.2% of cycles in 2010 to 77.3% of cycles in 2019.^2^ Thus, good quality embryos that are not transferred are most often cryopreserved for later use. However, many of these embryos are ultimately not used. In an analysis of more than 400 000 ART cycles in the US between 2004 and 2013, Christianson et al^3^ found that a total of 1 954 548 embryos had been cryopreserved and more than 1.2 million of those embryos had not been subsequently transferred. Patients must choose between discarding the embryos, donating them to the embryology laboratory for teaching purposes, donating them to another patient, or paying to store the embryos in perpetuity. Patients can feel conflicted about this decision, so continue paying for storage of cryopreserved embryos indefinitely.^4,5,6,7,8,9^

Surplus cryopreserved embryos also pose a dilemma for ART clinics. As the number of cryopreserved embryos increases, clinics are faced with logistical and financial strains in having to indefinitely store embryos while struggling to contact patients for their instruction on disposition.^10^ Recently, the Ethics Committee of the American Society for Reproductive Medicine updated its guidelines around unclaimed embryos, emphasizing the financial and decision challenges that surplus embryos raise for patients and ART programs.^11^

Finally, new issues have emerged regarding surplus embryos. There is great uncertainty among ART clinicians in the US as to how the US Supreme Court’s recent decision on Roe v. Wade will affect how they manage surplus embryos. With the ruling overturned, numerous states are considering “Personhood Legislation” that seeks to define the start of human life at fertilization. If this occurs, ART clinics may worry about potential legal risks involved in handling or discarding surplus embryos. These concerns may have substantial impacts on how ART is practiced, and ART clinics may desire to limit embryo creation due to potential legal implications.

One strategy to limit the number of surplus embryos is to limit the number of embryos created in the first place. This can occur by limiting the number of oocytes exposed to sperm and freezing or discarding the unexposed oocytes. Despite the recognized growing problem of surplus embryos and the option of oocyte vitrification, per current standards of care, clinics expose all oocytes to sperm (either with conventional insemination or intracytoplasmic sperm injection) in an ART cycle, because there currently is no validated method by which a clinic can determine how many oocytes to attempt to fertilize that will optimize patient outcomes. Of course, not all oocytes exposed to sperm result in transferable embryos and not all embryo transfers result in a live birth.

To answer the requests of patients and clinicians who seek to limit the number of surplus embryos created, we addressed the lack of evidence-based guidance in this area by developing a prediction algorithm according to patient and ART cycle characteristics. This prediction algorithm can aid clinicians in determining the optimal number of oocytes to expose to sperm. Optimal number is defined as enough oocytes exposed to sperm to preserve the chance of live birth while minimizing the number of surplus embryos created. Our hypothesis was that we could develop an algorithm that would provide a suitable estimate for the optimal number of oocytes to be exposed to sperm. This knowledge at the time of insemination would allow patients and clinicians to prioritize reducing the number of surplus embryos when that is an element of their decision process.

## Methods

This study was approved by the Amherst College institutional review board and the Society for Assisted Reproductive Technology (SART) Research Committee with a waiver of project-specific informed consent as secondary data analysis of an existing deidentified database posing minimal risk. This study followed the Transparent Reporting of a Multivariable Prediction Model for Individual Prognosis or Diagnosis (TRIPOD) reporting guideline. We used data from the national SART Clinical Outcome Reporting System (CORS). Full details about SART CORS data are included in Supplement 1. Patients who initiated their first stimulation cycle between January 1, 2014, and December 31, 2019, and had at least 1 oocyte retrieved were eligible for inclusion in the study. Cycles that used donor oocytes or gestational carriers were excluded. Cycles that intended to use or did use preimplantation genetic screening were also excluded.

### Statistical Analysis

The algorithm was developed in multiple stages: First, predict the day of transfer (day 3 or day 5). Among those with predicted day 3 transfer (eg, patients with a poor prognosis considering age or number of oocytes retrieved), recommend exposing all oocytes to sperm. Second, among those with day 5 predicted transfer, estimate 2 separate end points: (1) the expected proportion of retrieved oocytes that yield usable blastocysts among planned day 5 transfer cycles; and (2) the expected number of blastocysts needed for transfer for 1 live birth to occur among day 5 transfers.

A ratio of the predictions from (1) and (2) (the expected number of blastocysts needed divided by the expected proportion of retrieved oocytes that will become usable blastocysts) produced the prediction for number of oocytes to expose to sperm among expected day 5 transfers. For intracytoplasmic sperm injection (ICSI) cycles, this ratio was then multiplied by 0.70 to reflect the fact that, on average, 70.0% of retrieved oocytes are mature. The ceiling function was applied to the ratio (or the ratio × 0.70, for ICSI cycles) to yield a whole number for the final prediction.

To provide some intuition behind the ratio computation, consider this hypothetical example. Suppose that, for the typical patient with a good prognosis at a given clinic, the proportion of retrieved oocytes that become usable blastocysts is 40.0%, and that the number of blastocysts transferred across cycles for a single patient (either until a live birth occurs, or before treatment is stopped) is 4 or fewer blastocysts for most patients with a good prognosis. Then, one would compute that 10 oocytes should be exposed to sperm (4 blastocysts needed ÷ 0.40 rate = 10) to yield 4 usable blastocysts. The remaining mature oocytes can be vitrified. Our algorithm performs a similar computation, but instead of using the overall blastocyst rate and number of blastocysts transferred for a clinic, we aimed to obtain more precise estimates for a specific patient according to their individual characteristics. Clean and complete data for this analysis were available in February 2022 and analyses were conducted from February to June 2022. R version 4.1.0 (R Project for Statistical Computing) was used for all analyses.^12^

#### Covariates Considered

Given that this prediction tool is intended to be used before an attempt to fertilize oocytes is made, only patient and cycle characteristics known at that point of treatment were considered as predictors in the models. Four sets of potential predictors were considered for each end point, and the final covariate set was chosen as the minimal set that meaningfully increased predictive performance (see eMethods 2 in Supplement 1 for the complete set of covariates considered). The final models included female patient age group, state where the clinic is located, anti-mullerian hormone (AMH) level, diminished ovarian reserve diagnosis, and the number of oocytes retrieved.

#### Training and Testing Sets

The SART CORS data was randomly separated at the patient-level into equal-sized training and testing data sets. The retrievals and transfers corresponding to patients in the training set were used to develop the models. The retrievals and transfers corresponding to patients in the testing set were used to evaluate how well the models performed in making predictions for new patients.

#### Model 1: Day of Transfer

It is standard practice in most ART clinics to perform a fresh embryo transfer 5 days after fertilization, at which time some percentage of embryos should become blastocysts, which contain about 100 cells, and cells have differentiated into an inner cell mass, which becomes the fetus, and trophectoderm cells, which become the placenta. Transfers that occurred on day 5 or later were included as day 5 (blastocyst) transfers. Some ART practices may also consider an embryo transfer on 2, 3, or 4 days after fertilization for patients with a poor prognosis (those who are expected to have a low number of eggs or embryos or when age is related to a lower likelihood of live birth). These transfers were included as day 3 (embryo) transfers. A logistic regression model was used to compute the probability of a day 5 transfer, and a probability threshold of 0.75 was chosen to classify predictions as day 3 or day 5 transfers. An explanation regarding the threshold decision, as well as the operating characteristics of different thresholds and models considered, are included in the eMethods, eFigure 1, and eFigure 2 in Supplement 1.

#### Model 2: Proportion of Retrieved Oocytes That Become Usable Blastocysts

The proportion of retrieved oocytes that become usable blastocysts was computed as the sum of the number of blastocysts transferred and the number of blastocysts frozen divided by the number of retrieved oocytes. Criteria for blastocyst cryopreservation includes degree of expansion (blastocyst size) and quality scores of the inner cell mass (the cells that develop into the fetus) and trophectoderm (the cells that develop into the placenta). The number of mature oocytes and the number of oocytes exposed to sperm are not data available in SART CORS. The operating characteristics of the models considered in estimating this proportion, including the final model, are included in the eMethods and eTable 1 in Supplement 1.

#### Model 3: Number of Blastocysts Transferred

The number of blastocysts transferred for 1 live birth to occur was defined at the patient level by summing the total number of blastocysts transferred across a patient’s treatment cycles until the first live birth was observed. Patients who had blastocysts transferred but no live birth observed were considered right-censored. Several different survival models were considered to predict the number of blastocysts transferred (the time) until 1 live birth (the event) occurred (eMethods in Supplement 1). Final predictions were derived as the median number of blastocysts estimated from an accelerated failure time survival model.

## Results

Among 311 237 patients aged 18 to 45 years old who initiated their first stimulation cycle between January 1, 2014, and December 31, 2019, and had at least 1 oocyte retrieved, 410 719 oocyte retrieval cycles produced 460 577 embryo transfer cycles. The median (IQR) age at stimulation cycle start was 35 (29-32) years and the median (IQR) number of oocytes retrieved was 10 (6-17) (Table 1). The most common reasons for ART were ovulation disorders (154 612 [37.6%]) and male factor infertility (142 149 [34.6%]). The majority of embryo transfers (365 630 [73.2%]) occurred on day 5 or later, and 167 837 (69.7%) day 5 transfer or freeze cycles had blastocysts, rather than earlier stage embryos, frozen.

**Table 1.** Patient and Cycle Characteristics Among 311 237 Patients Who Initiated Their First Stimulation Cycle Between January 1, 2014, and December 31, 2019, at a Clinic That Reports to the Society for Assisted Reproductive Technologies Clinical Outcome Reporting System, Stratified by Day of Transfer or Freeze

Characteristic	No. (%)	
	Day 3	Day 5
No. of oocyte retrieval cycles	89 769	240 644
Patient age, y		
Median (IQR)	37.0 (33.0-40.0)	34.0 (31.0-37.0)
<32	13 123 (14.6)	73 758 (30.7)
32-34	15 245 (17.0)	62 297 (25.9)
35-37	19 535 (21.8)	52 751 (21.9)
38-40	21 138 (23.5)	33 582 (14.0)
41-42	11 924 (13.3)	11 976 (5.0)
>42	8804 (9.8)	6280 (2.6)
Body mass index^a^		
<18.5	1801 (2.0)	4434 (1.8)
18.5 to 24.9	38 654 (43.1)	97 962 (40.7)
25.0 to 29.9	19 577 (21.8)	52 695 (21.9)
30.0 to 34.9	9773 (10.9)	27 682 (11.5)
35 to 39.9	5127 (5.7)	14 638 (6.1)
>40	3124 (3.5)	7351 (3.1)
Unknown	11 713 (13)	35 882 (15)
Gravidity		
0	40 963 (45.6)	121 060 (50.3)
1	23 488 (26.2)	60 672 (25.2)
2	12 677 (14.1)	30 479 (12.7)
≥3	12 303 (13.7)	27 698 (11.5)
Unknown	338 (0.4)	735 (0.3)
Day 3 follicle stimulating hormone, mIU/mL		
≤10	50 687 (56.5)	142 395 (59.2)
>10	14 834 (16.5)	17 823 (7.4)
Unknown	24 248 (27.0)	80 426 (33.4)
Latest anti-mullerian hormone, ng/mL		
<1	23 626 (26.3)	23 893 (9.9)
1-<4	27 366 (30.5)	81 585 (33.9)
≥4	7501 (8.4)	50 902 (21.2)
Unknown	31 276 (34.8)	84 264 (35.0)
Male factor infertility	30 292 (33.7)	89 579 (37.2)
Tubal factor infertility	13 077 (14.6)	36 779 (15.3)
Ovulation factor infertility	37 607 (41.9)	79 366 (33.0)
Endometriosis	8288 (9.2)	21 050 (8.7)
Uterine factor infertility	4452 (5.0)	11 860 (4.9)
Diminished ovarian reserve	37 855 (42.2)	42 003 (17.5)
Unexplained infertility	11 592 (12.9)	39 750 (16.5)
No. of oocytes retrieved, median (IQR)	7 (4-11)	13 (9-20)
Intracytoplasmic sperm injection cycle	70 421 (78.4)	172 427 (71.7)
Proportion of retrieved oocytes that yielded usable blastocysts	NA	0.29 (0.18-0.43)
Any blastocysts frozen	NA	167 837 (69.7)
No. of blastocysts frozen, among cycles with any frozen	NA	2.0 (4.0-6.0)
Embryo transfers		
No.	94 947	365 630
No. of embryos transferred, median (IQR)	2 (2-3)	1 (1-2)
Live birth	23 126 (24.4)	158 835 (43.4)

^a^ Body mass index is calculated as weight in kilograms divided by height in meters squared.

### Stage 1: Day of Transfer

Younger age, higher AMH level, a higher number of oocytes retrieved, and no diagnosis of diminished ovarian reserve were associated with increased probability of a day 5 transfer. For instance, among oocyte retrievals in the study population, patients were predicted to have a day 5 transfer at the following rates: 83.9% of patients younger than 32 years, 75.2% of patients 32 to 34 years, 58.2% of patients 35 to 37 years, 33.8% of patients 38 to 40 years, 16.5% of patients 41 to 42 years, and 7.9% of patients older than 42 years (Figure). Similarly, 10 358 (14.8%) patients with an AMH level less than 1 ng/mL vs 58 999 (90.8%) patients with an AMH level 4 ng/mL or greater were predicted to have a day 5 transfer.

**Figure.**
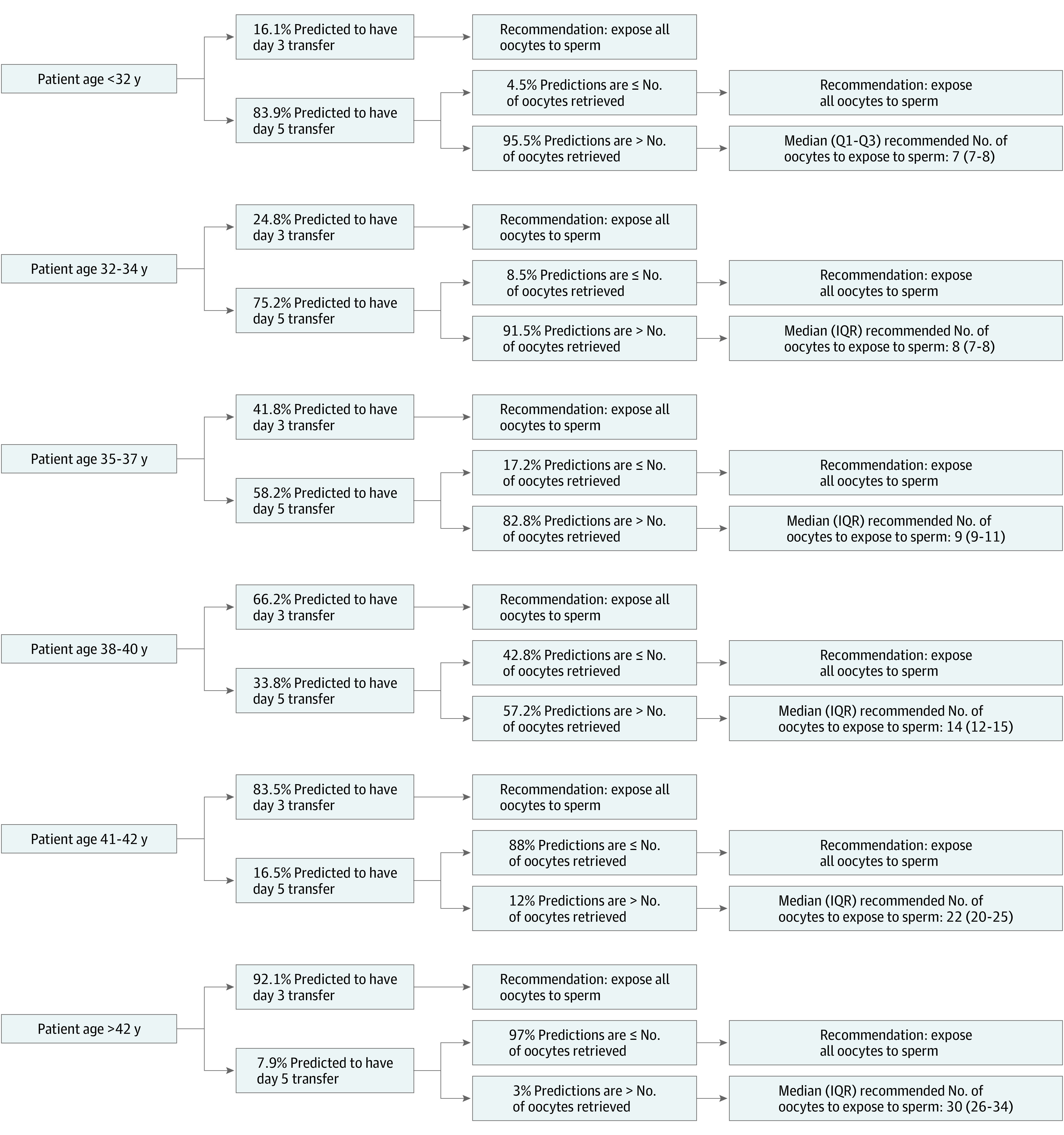
Flowchart of the Recommendations for the Predicted Number of Oocytes to Expose to Sperm Among oocyte retrievals for patients who initiated a first stimulation cycle at a Society for Assisted Reproductive Technologies Clinical Outcome Reporting System member clinic between January 1, 2014, and December 31, 2019.

### Stage 2: Among Cycles Predicted to Have Day 5 Transfer or Freeze

#### End Point 1. Proportion of Retrieved Oocytes That Become Usable Blastocysts

The median (IQR) prediction for the proportion of retrieved oocytes that will become usable blastocysts was 0.30 (0.2-0.33). The median (IQR) decreased with increasing age, from 0.31 (0.28-0.33) among patients younger than 32 years to 0.21 (0.16-0.25) among patients older than 42 years.

#### End Point 2: Number of Blastocysts Transferred for 1 Live Birth to Occur

The predicted number of blastocysts transferred to result in 1 live birth increased with increasing age. The median (IQR) prediction among patients expected to have a day 5 transfer was 2.1 (1.9-2.2) blastocysts for patients younger than 32 years, 2.2 (2.0-2.3) for patients aged 32 to 34 years, 2.5 (2.4-2.7) for patients aged 35 to 37 years, 3.3 (3.1-3.6) for patients aged 38 to 40 years, 5 (4.7-5.4) for patients aged 41 to 42 years, and 6.8 (6.4-7.3) for patients older than 42 years.

### Final Prediction

Overall, 43.4% of oocyte retrievals in the study population were predicted to be day 3 transfers, and on that basis, exposing all oocytes to sperm would be recommended. The remaining 57.6% of cycles predicted as day 5 transfers proceeded to the next stage to estimate the number of oocytes to expose to sperm. Among the patients expected to have a day 5 transfer, the median (IQR) numbers of oocytes to expose to sperm for 1 live birth were 7 (7-8) oocytes for patients younger than 32 years, 8 (7-8) for patients 32 to 34 years, and 9 (9-11) for patients 35 to 37 years old (Table 2).

**Table 2.** Recommendations for the Predicted Number of Oocytes to Expose to Sperm, by Age Group, for Oocyte Retrievals Among Patients Who Initiated a First Stimulation Cycle at a Society for Assisted Reproductive Technologies Clinical Outcome Reporting System Member Clinic Between January 1, 2014, and December 31, 2019

Age group, y	Cycles recommended to expose all oocytes to sperm, No. (%)^a^	Cycles recommended to expose fewer than all oocytes to sperm^b^	
		No. (%)	Patient-specific recommended No. of oocytes to expose to sperm, median (IQR)
<32	19 373 (19.9)	77 761 (80.1)	7 (7-8)
32-34	27 834 (31.2)	61 248 (68.8)	8 (7-8)
35-37	45 346 (51.8)	42 260 (48.2)	9 (9-11)
38-40	59 223 (80.6)	14 211 (19.4)	14 (12-15)
41-42	35 819 (98.0)	724 (2.0)	22 (20-25)
>42	26 856 (99.8)	64 (0.2)	30 (26-34)

^a^ Recommended to expose all oocytes to sperm either because of a predicted day 3 transfer or because of a predicted day 5 transfer where the predicted number of oocytes to expose to sperm was greater than or equal to the number of oocytes retrieved. See Figure for the breakdown of reason by age group.

^b^ Recommended to expose fewer than all oocytes to sperm because of a predicted day 5 transfer and the predicted number of oocytes to expose to sperm was less than the number of oocytes retrieved.

Overall, 196 268 (47.8%) oocyte retrievals in the study population indicated attempting to fertilize fewer oocytes than the number of oocytes retrieved. This number varied by age, being more than 77 761 (80.0%) for patients younger than 32 years old and 724 (<2.0%) for patients 41 to 42 years old. If all patients in the test set had used this tool, 990 394 fewer oocytes would have been exposed to sperm.

We have developed an interactive website^13^ that clinicians and patients can use to compute the recommended number of oocytes to expose to sperm according to this algorithm. This interactive tool additionally accounts for the number of desired children to result from a single oocyte stimulation cycle and single gestation pregnancy/pregnancies by multiplying the final prediction by the number of desired children. An example of 2 patient use cases of the website is included in eFigure 3 in Supplement 1.

## Discussion

In this diagnostic study, to support patients and clinicians who wish to limit the creation of surplus embryos, we developed an evidence-based prediction tool to determine how many oocytes should be exposed to sperm to optimize live birth rates but minimize the number of surplus embryos. The results suggest that fewer oocytes than the number retrieved could be exposed to sperm to get 1 live birth in more than half of ART stimulation cycles among patients younger than 38 years old and in about 20.0% of stimulation cycles among patients 38 to 40 years old. For nearly all patients older than 40 years, all oocytes retrieved should be exposed to sperm.

To our knowledge, there is no prior literature attempting to determine how to limit the number of oocytes exposed to sperm to reduce the number of surplus embryos. Recent studies examining predictors of blastocyst formation rate identified similar predictors as our blastocyst model, including female patient age and number of (mature) oocytes.^14,15,16^ However, each of those studies was based on data from a single clinic with a fairly small sample size (107, 520, and 4205 patients). Robertson et al^15^ developed a model to predict a patient’s likelihood of having embryos available to transfer or freeze on day 5, similar to our first model end point. They identified number of oocytes retrieved, patient age, and infertility diagnosis as important predictors, which is consistent with the predictors we identified in our model.

ART practitioners and patients may be hesitant to use the prediction tool given the novelty of the concept of attempting to fertilize fewer oocytes. In the past, single embryo transfer (SET) recommendations were met with much resistance when first suggested as a way to limit the number of multiple pregnancies. Opposition was strong due to perceived concerns that SET would lower live birth rates, and the preference for multiple pregnancy over no pregnancy. However, a growing body of research demonstrated the effectiveness of SET and with time, practices began to change such that 77.3% of transfers in the US in 2019 were SET.^2^ Similarly, our hope in developing this prediction tool is that it will address the immediate needs of patients and clinicians who are concerned about creating surplus embryos. Our evidence-based estimate for the number of oocytes that should be exposed to sperm will reduce the number of excess embryos requiring long term storage. Ideally, usage of this tool will motivate research that identifies oocyte characteristics associated with higher quality embryos, moving us toward second-generation science which could allow an algorithm to predict not only how many oocytes should be exposed to sperm, but which oocytes to expose to sperm.

### Strengths and Limitations

Our study has several strengths, including the use of a large, national database that captures more than 90.0% of ART cycles in the US. The SART CORS registry includes a large number of variables, and we were able to consider a number of known and potentially important prefertilization predictors in our models. Due to the large sample size, we were able to incorporate many predictors in the models without encountering convergence errors.

Our study has some limitations, including lack of information on the number of mature oocytes. In cycles that use intracytoplasmic sperm injection, only mature oocytes are injected with sperm. The proportion of retrieved oocytes that become usable blastocysts provides an underestimate of the proportion of mature oocytes that become usable blastocysts and leads to overestimating the number of oocytes to expose to sperm in ICSI cycles. We attempted to account for this by multiplying the ratio by 0.70 in ICSI cycles. However, having information on the number of mature oocytes retrieved would have provided a more accurate estimate.

## Conclusions

Patients and ART clinics are caught in a tangled web of quickly changing federal and state laws. It is unclear whether patient autonomy for personal decisions regarding using, storing, and/or discarding embryos will be possible. The current political environment may force transformation of ART practices in which the number of embryos created must be minimized to avoid discarding or abandoning embryos. Clinicians will have to consider the clinical success, logistic, and financial practice of multiple rounds of egg thawing, fertilization, and transfer against the legal implications and loss of personal autonomy regarding surplus embryos. Our diagnostic study allows patients and clinicians a tool to minimize embryo creation if they deem it appropriate for their practice setting and personal preference.

## Notes

Supplement 1. **eMethods 1.** Additional Information Regarding SARTCORS Methods **eMethods 2.** Model Selection and Operating Characteristics **eFigure 1.** Receiver Operating Characteristic (ROC) Curve for 4 Logistic Regression Models With Predictor Sets A (Red), B (Orange), C (Green), and D (Blue) **eFigure 2.** Prediction Accuracy, Sensitivity, Specificity, Positive Predictive Value, and Negative Predictive Value, by Training and Testing Set, Among Select Models With Predictor Set (B) Fit for Predicting Day of Transfer **eTable 1.** The Mean Absolute Error and Root Mean Squared Error of Different Predictor Sets in Random Forest Algorithms to Predict Proportion of Useable Blastocysts Among Retrieved Oocytes, by Training and Testing Set **eTable 2.** Clinic Location Among 311 237 Women Who Initiated Their First Stimulation Cycle Between January 1, 2014, and December 31, 2019, at a Clinic That Reports to the Society for Assisted Reproductive Technologies Clinical Outcome Reporting System (SART CORS), Stratified by Day of Transfer or Freeze **eFigure 3.** Example of 2 Patient Use Cases of the Interactive Website

Supplement 2.
Data Sharing Statement
