# Supplementary material for: Development of a Model to Estimate the Optimal Number of Oocytes to Attempt to Fertilize During Assisted Reproductive Technology Treatment
Source: JAMA Netw Open. 2023 Jan 3;6(1):e2249395. doi: 10.1001/jamanetworkopen.2022.49395 (PMC9857446; doi:10.1001/jamanetworkopen.2022.49395)
Supplement: Supplement 1. — eMethods 1. Additional Information Regarding SARTCORS Methods eMethods 2. Model Selection and Operating Characteristics eFigure 1. Receiver Operating Characteristic (ROC) Curve for 4 Logistic Regression Models With Predictor Sets A (Red), B (Orange), C (Green), and D (Blue) eFigure 2. Prediction Accuracy, Sensitivity, Specificity, Positive Predictive Value, and Negative Predictive Value, by Training and Testing Set, Among Select Models With Predictor Set (B) Fit for Predicting Day of Transfer eTable 1. The Mean Absolute Error and Root Mean Squared Error of Different Predictor Sets in Random Forest Algorithms to Predict Proportion of Useable Blastocysts Among Retrieved Oocytes, by Training and Testing Set eTable 2. Clinic Location Among 311 237 Women Who Initiated Their First Stimulation Cycle Between January 1, 2014, and December 31, 2019, at a Clinic That Reports to the Society for Assisted Reproductive Technologies Clinical Outcome Reporting System (SART CORS), Stratified by Day of Transfer or Freeze eFigure 3. Example of 2 Patient Use Cases of the Interactive Website [file jamanetwopen-e2249395-s001.pdf]

Supplemental Online Content

Correia KFB, Missmer SA, Weinerman R, Ginsburg ES, Rossi BV. Development of a model to estimate the optimal number of oocytes to attempt to fertilize during assisted reproductive technology treatment. *JAMA Netw Open*. 2023;6(1):e2249395.
doi:10.1001/jamanetworkopen.2022.49395

eMethods 1. Additional Information Regarding SARTCORS Methods

eMethods 2. Model Selection and Operating Characteristics

eFigure 1. Receiver Operating Characteristic (ROC) Curve for 4 Logistic Regression Models With Predictor Sets A (Red), B (Orange), C (Green), and D (Blue)

eFigure 2. Prediction Accuracy, Sensitivity, Specificity, Positive Predictive Value, and Negative Predictive Value, by Training and Testing Set, Among Select Models With Predictor Set (B) Fit for Predicting Day of Transfer

eTable 1. The Mean Absolute Error and Root Mean Squared Error of Different Predictor Sets in Random Forest Algorithms to Predict Proportion of Useable Blastocysts Among Retrieved Oocytes, by Training and Testing Set

eTable 2. Clinic Location Among 311 237 Women Who Initiated Their First Stimulation Cycle Between January 1, 2014, and December 31, 2019, at a Clinic That Reports to the Society for Assisted Reproductive Technologies Clinical Outcome Reporting System (SART CORS), Stratified by Day of Transfer or Freeze

eFigure 3. Example of 2 Patient Use Cases of the Interactive Website

This supplemental material has been provided by the authors to give readers additional information about their work.

© 2023 Correia K et al. *JAMA Network Open.*

eMethods 1. Additional Information Regarding SARTCORS Methods

The data used for this study were obtained from the SART Clinic Outcome Reporting System (SART CORS). Data were collected through voluntary submission, verified by SART, and reported to the Centers for Disease Control and Prevention (CDC) in compliance with the Fertility Clinic Success Rate and Certification Act of 1992 (Public Law 102-493). SART maintains HIPAA-compliant business associates agreements with reporting clinics. In 2004, following a contract change with the CDC, SART gained access to the SART CORS data system for the purposes of conducting research. In 2017, 82% of all assisted reproductive technology (ART) clinics in the United States were SART members.[1]

The data in the SART CORS are validated annually with 7-10% of clinics receiving on-site visits for chart review based on an algorithm for clinic selection. During each visit, data reported by the clinic were compared with information recorded in patients' charts. In 2019, records for 2,014 cycles at 34 clinics were randomly selected for full validation, along with 213 fertility preservation cycles selected for partial validation. The full validation included review of 1,300 cycles for which a pregnancy was reported. Nine out of eleven data fields selected for validation were found to have discrepancy rates of ≤5%.[2] The exceptions were the diagnosis field, which, depending on the diagnosis, had a discrepancy rate between 2.5% and 17.8%, and the start date, which had a 8.4% discrepancy rate.[2] Obstetrical outcomes from Massachusetts ART records during 2004-2008 have been validated to have >95% agreement with vital records.[3]

© 2023 Correia K et al. *JAMA Network Open.*

eMethods 2. Model Selection and Operating Characteristics

For each of the endpoints, four sets of predictors were considered: (A) female patient age category (<32, 32-34, 35-37, 38-40, 41-42, >42 years) and state where the clinic is located; (B) female patient age category, clinic location, last anti-Mullerian hormone (AMH) level (< 1 ng/ml, 1-4 ng/ml, >4 ng/ml), diminished ovarian reserve diagnosis, number of oocytes retrieved; (C) female patient age category, clinic location, AMH level, diminished ovarian reserve diagnosis, number of oocytes retrieved, BMI group, gravidity, parity, maximum follicle stimulating hormone (FSH) level (≤ 10 mIU/ml, > 10 mIU/ml), male infertility diagnosis, tubal factor infertility diagnosis, endometriosis, uterine factor infertility diagnosis, ovulatory infertility diagnosis, and an unexplained infertility diagnosis; and (D) female patient age category, clinic location, male partner age, AMH level, diminished ovarian reserve diagnosis, number of oocytes retrieved, body mass index (kg/m^2) group, gravidity (0, 1, 2, 3+), parity (0, 1, 2, 3+), FSH level, male infertility diagnosis, tubal factor infertility diagnosis, endometriosis, uterine factor infertility diagnosis, ovulatory infertility diagnosis, and an unexplained infertility diagnosis, low sperm motility, low sperm morphology.

The largest covariate set (set D) included all potentially relevant predictors that are measured prior to attempting to fertilize oocytes; factors measured after that timepoint won't be known at time of using the prediction tool and thus were eliminated from consideration. We considered reduced sets of covariates because (1) there was reason to believe the addition of certain factors above and beyond age, clinic state, and ovarian reserve parameters likely wouldn't meaningfully increase the accuracy of the prediction; and (2) simpler models would make for a smoother and more straightforward user interface for the online prediction tool, so we wanted to include the minimal set of necessary covariates.

Model 1: Predicting Day of Transfer

Logistic regression models and random forest classification algorithms were fit to the data to predict day of transfer (day 3 or day 5). The random forest algorithms output a classification (day 3 or day 5), whereas the logistic regression models output a predicted probability of a day 5 transfer. We classified predictions from the logistic regression models using different probability thresholds between 0.40 and 0.80 (e.g., if the predicted probability was ≥ 0.5, classify the observation as a day 5 transfer, and if the predicted probability was < 0.5, classify the observation as a day 3 transfer).

We compared models and probability thresholds using the following measures:

- Accuracy: the proportion of observations that were correctly predicted
  - (number of true day 5 transfers predicted to be a day 5 transfer + number of true day 3 transfers predicted to be a day 3 transfer)/(total number of transfers)
- Sensitivity: the proportion of true day 5 transfers that were correctly predicted
  - (number of true day 5 transfers predicted to be a day 5 transfer)/(number of true day 5 transfers)
- Specificity: the proportion of true day 3 transfers that were correctly predicted
  - (number of true day 3 transfers predicted to be a day 3 transfer)/(number of true day 3 transfers)
- Positive predictive value: among those predicted to have a day 5 transfer, the proportion who actually have a day 5 transfer
  - (number of true day 5 transfers predicted to be a day 5 transfer)/(number predicted to have day 5 transfer)
- Negative predictive value: among those predicted to have a day 3 transfer, the proportion who actually have a day 3 transfer
  - (number of true day 3 transfers predicted to be a day 3 transfer)/(number predicted to have day 3 transfer)

The specificity under the random forest algorithms were all below 40%, and thus the random forests were deemed unsuitable. Because day 3 transfers are poorer prognosis patients (and will be recommended to expose all oocytes to sperm), we wanted to be cautious of "false positives" which would incorrectly label a day 3 transfer as a day 5 transfer.

© 2023 Correia K et al. *JAMA Network Open.*

eFigure 1 compares the receiver operating characteristic (ROC) curves for the four logistic regression models and suggests that the model with predictors set B (and an area under the curve (AUC) of 0.813, 95% CI: 0.811, 0.815) is better than the model with predictors set A (AUC: 0.720, 95% CI: 0.718, 0.723) and just as good as the models with predictors set C (AUC: 0.816, 95% CI: 0.814, 0.818) and D (AUC: 0.818, 95% CI: 0.816, 0.820).

eFigure 2 compares the accuracy, sensitivity, specificity, positive predictive value, and negative predictive value across models and different probability thresholds. Based on these operating characteristics on the testing set of patients, and their meaning in this particular context, the final model was chosen as the logistic regression model with predictors in set B and a probability threshold of 0.75. Although predictive accuracy on the testing set was maximized at a probability threshold of 0.50 with a value >80%, the specificity was quite poor at that threshold (31%). Using a probability threshold of 0.75, the predictive accuracy on the testing set was highest while also constraining both the sensitivity and specificity to be ≥ 65%. In particular, with regards to the testing set, the predictive accuracy was 76.5% (95% CI: 76.3, 76.6), sensitivity was 78.2% (95% CI: 78.1, 78.4), specificity was 69.6% (95% CI: 69.4, 69.8), positive predictive value was 90.8% (95% CI: 90.7, 91.0), and negative predictive value was 45.3% (95% CI: 45.1, 45.5).

Model 2: Predicting Proportion of Useable Blastocysts

The distribution of the observed proportion of useable blastocysts was skewed and multi-modal. In contrast to linear regression and beta regression (which is often used when the outcome is a proportion), random forest algorithms are non-parametric. They have no formal distributional assumptions and can handle non-linearities and interactions. Thus, we used random forests to predict the proportion of useable blastocysts. eTable 1 displays the mean absolute error (MAE) and the root mean squared error (RMSE) for each set of predictors by testing and training set. Lower values indicate better predictive ability.

The final predictor set B was chosen based on the MAE and RMSE on the testing set, which is indicative of the algorithm's performance on "new" patients.

Model 3: Predicting Number of Embryos Needed for Transfer to Result in One Live Birth

The number of blastocysts transferred for one live birth to occur was defined at the patient-level by summing the total number of blastocysts transferred across a patient's treatment cycles until the first live birth was observed. For example, suppose a patient had one blastocyst transferred in the first cycle which did not result in a viable pregnancy and then two blastocysts transferred in a second cycle which resulted in a singleton live birth. The "time" here would be three because three total blastocysts were transferred to result in one live birth.

Cox proportional hazard models with predictor sets A, B, C, and D were initially fit to the data, but tests of proportionality demonstrated that the proportional hazard assumption was not reasonable for most of the covariates.[4] Accelerated-failure time models were then fit with log-logistic distribution which allows for non-monotone hazard (the hazard can increase and then decrease over time).[5] The concordance index indicated similar concordance (0.64) for predictor sets B, C, and D, and thus predictor set B was used as the final predictor set.

References

1. Centers for Disease Control and Prevention. 2017 Assisted Reproductive Technology Fertility Clinic Success Rates Report. Atlanta, GA: 2019.

© 2023 Correia K et al. *JAMA Network Open.*

2. Centers for Disease Control and Prevention. 2019 Assisted Reproductive Technology Fertility Clinic and National Summary Report [Internet]. 2022 [cited 2022 Feb 7]. Available from: https://www.cdc.gov/art/reports/2019/pdf/2019-Report-ART-Fertility-Clinic-National-Summary-h.pdf

3. Stern JE, Gopal D, Liberman RF, Anderka M, Kotelchuck M, Luke B. Validation of birth outcomes from the Society for Assisted Reproductive Technology Clinic Outcome Reporting System (SART CORS): population-based analysis from the Massachusetts Outcome Study of Assisted Reproductive Technology (MOSART). Fertility and Sterility 2016;106(3):717-722.e2.

4. Grambsch PM, Therneau TM. Proportional hazards tests and diagnostics based on weighted residuals. Biometrika 1994;81(3):515–26.

5. Wei LJ. The accelerated failure time model: A useful alternative to the cox regression model in survival analysis. Statistics in Medicine 1992;11(14–15):1871–9.

© 2023 Correia K et al. *JAMA Network Open.*

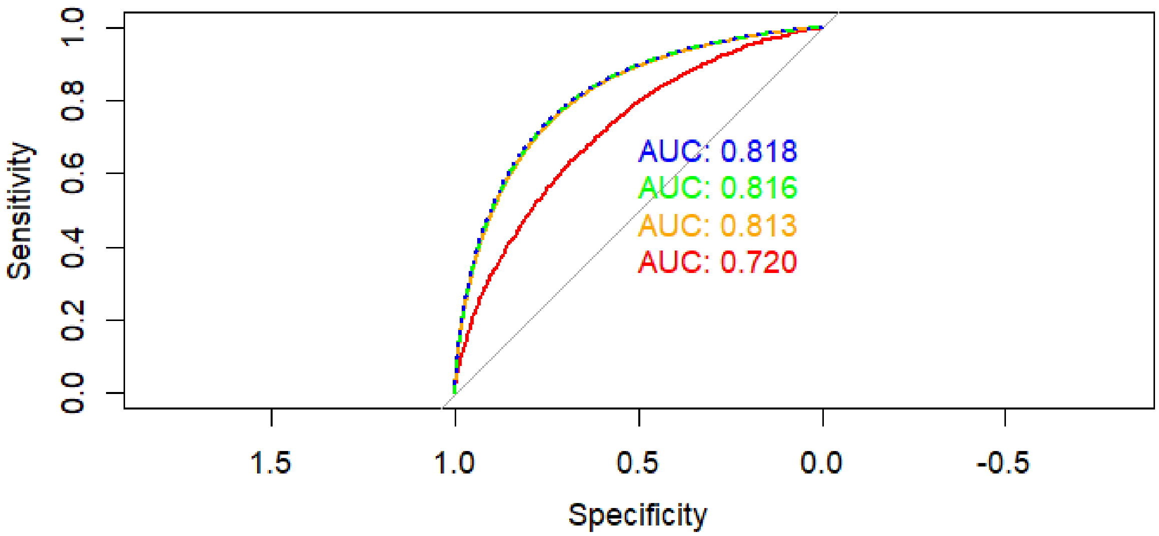

eFigure 1. Receiver Operating Characteristic (ROC) Curve for 4 Logistic Regression Models With Predictor Sets A (Red), B (Orange), C (Green), and D (Blue).

© 2023 Correia K et al. *JAMA Network Open.*

eFigure 2. Prediction Accuracy, Sensitivity, Specificity, Positive Predictive Value, and Negative Predictive Value, by Training and Testing Set, Among Select Models With Predictor Set (B) Fit for Predicting Day of Transfer.

Predictor set (B) included age group, clinic state, AMH level, diminished ovarian reserve diagnosis, number of oocytes retrieved.[1]

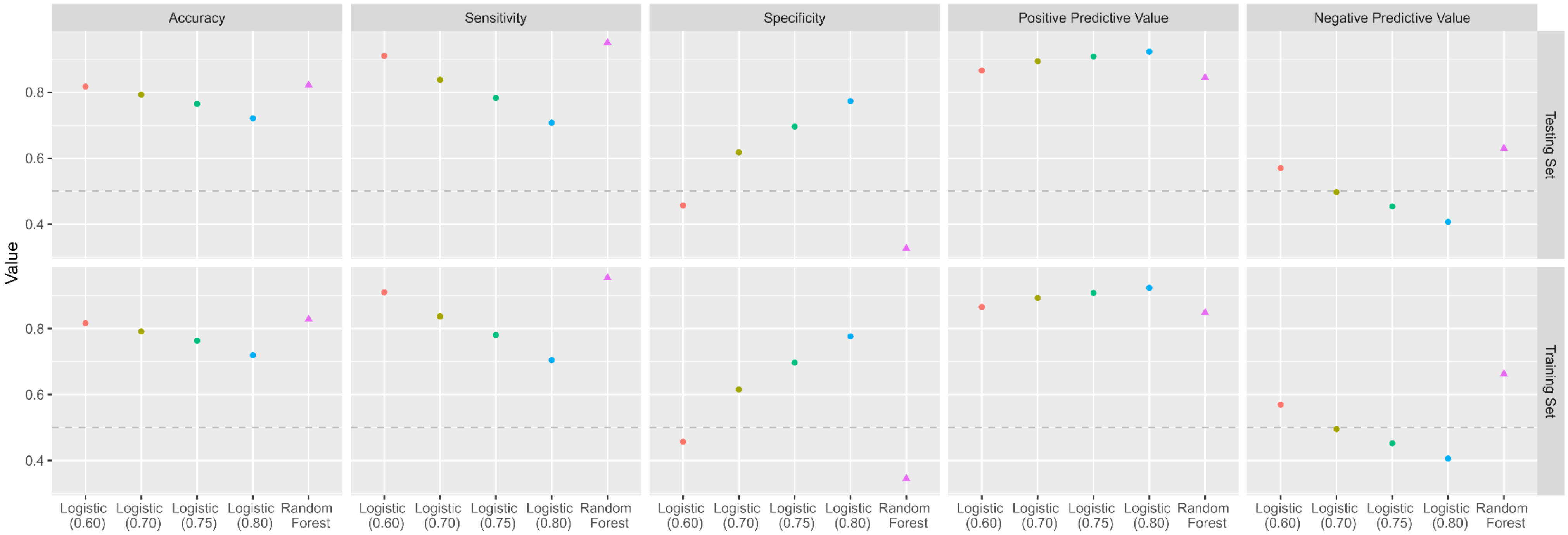

[1] 95% confidence intervals for each measure are ≤ 0.002 units from the point estimate, spanning less than the size of the point on the figure, and thus are not indicated in the figure. For instance, the accuracy in the testing set for the logistic regression model with a probability threshold of 0.60 is 0.817 (95% CI: 0.816, 0.819).

© 2023 Correia K et al. *JAMA Network Open.*

eTable 1. The Mean Absolute Error and Root Mean Squared Error of Different Predictor Sets[1] in Random Forest Algorithms to Predict Proportion of Useable Blastocysts Among Retrieved Oocytes, by Training and Testing Set.

	Training Set	Testing Set
Mean Absolute Error		
Predictor set A	0.15	0.15
Predictor set B	0.13	0.14
Predictor set C	0.11	0.14
Predictor set D	0.10	0.14
Root Mean Squared Error		
Predictor set A	0.19	0.19
Predictor set B	0.16	0.17
Predictor set C	0.14	0.17
Predictor set D	0.12	0.17

[1] Predictor set A included age group and clinic state; Predictor set B included age group, clinic state, AMH level, diminished ovarian reserve diagnosis, and number of oocytes retrieved; Predictor set C included all predictors in predictor set B plus BMI group, gravidity, parity, FSH level, male infertility diagnosis, tubal factor infertility diagnosis, endometriosis, uterine factor infertility diagnosis, ovulatory infertility diagnosis, and an unexplained infertility diagnosis; Predictor set D included all predictors in predictor set C plus low sperm motility and low sperm morphology.

© 2023 Correia K et al. *JAMA Network Open.*

eTable 2. Clinic Location Among 311 237 Women who Initiated Their First Stimulation Cycle Between January 1, 2014, and December 31, 2019 at a Clinic That Reports to the Society for Assisted Reproductive Technologies Clinical Outcome Reporting System (SART CORS), Stratified by Day of Transfer or Freeze.

Clinic location	Day 3<br>N = 89,769[1]	Day 5<br>N = 240,644[1]
AL	477 (0.5%)	1,825 (0.8%)
AZ	1,915 (2.1%)	2,514 (1.0%)
CA	10,086 (11.2%)	19,129 (7.9%)
CO	520 (0.6%)	1,551 (0.6%)
CT	1,431 (1.6%)	6,139 (2.6%)
DC	629 (0.7%)	917 (0.4%)
DE	15 (0.0%)	574 (0.2%)
FL	2,373 (2.6%)	9,720 (4.0%)
GA	1,774 (2.0%)	4,646 (1.9%)
HI	675 (0.8%)	1,591 (0.7%)
IA	167 (0.2%)	2,422 (1.0%)
IL	7,488 (8.3%)	17,818 (7.4%)
IN	638 (0.7%)	4,214 (1.8%)
KS	117 (0.1%)	1,902 (0.8%)
KY	64 (0.1%)	399 (0.2%)
LA	159 (0.2%)	2,258 (0.9%)
MA	9,425 (10.5%)	18,826 (7.8%)
MD	2,671 (3.0%)	18,914 (7.9%)
MI	3,546 (4.0%)	5,326 (2.2%)
MN	1,128 (1.3%)	5,208 (2.2%)
MO	1,750 (1.9%)	2,646 (1.1%)
MS	37 (0.0%)	330 (0.1%)
NC	1,151 (1.3%)	6,440 (2.7%)
NE	11 (<0.0%)	1,585 (0.7%)
NJ	3,084 (3.4%)	10,962 (4.6%)

© 2023 Correia K et al. *JAMA Network Open.*

Clinic location	Day 3<br>N = 89,769[1]	Day 5<br>N = 240,644[1]
NV	69 (0.1%)	1,476 (0.6%)
NY	18,307 (20.4%)	27,197 (11.3%)
OH	3,193 (3.6%)	7,950 (3.3%)
OK	543 (0.6%)	1,834 (0.8%)
OR	99 (0.1%)	1,254 (0.5%)
Other	2,732 (3.0%)	4,755 (2.0%)
PA	3,565 (4.0%)	7,879 (3.3%)
SC	684 (0.8%)	2,830 (1.2%)
TN	626 (0.7%)	2,269 (0.9%)
TX	4,190 (4.7%)	19,645 (8.2%)
UT	510 (0.6%)	4,451 (1.8%)
VA	1,686 (1.9%)	3,391 (1.4%)
VT	427 (0.5%)	488 (0.2%)
WA	1,261 (1.4%)	5,104 (2.1%)
WI	546 (0.6%)	2,265 (0.9%)

[1] n (%)

© 2023 Correia K et al. *JAMA Network Open.*

eFigure 3. Example of 2 Patient Use Cases of the Interactive Website.

The hypothetical patients in both cases are at a clinic in Ohio and have 15 eggs retrieved. They desire one child and currently have no children. Panel A) displays the inputs and outputs for such a patient who is 30 years old, has not been diagnosed with diminished ovarian reserve, and has an anti-mullerian hormone level of 2.5 ng/mL. Panel B) displays the inputs and outputs for such a patient who is 38 years old, has been diagnosed with diminished ovarian reserve, and has an anti-mullerian hormone level of 0.9 ng/mL.

A.

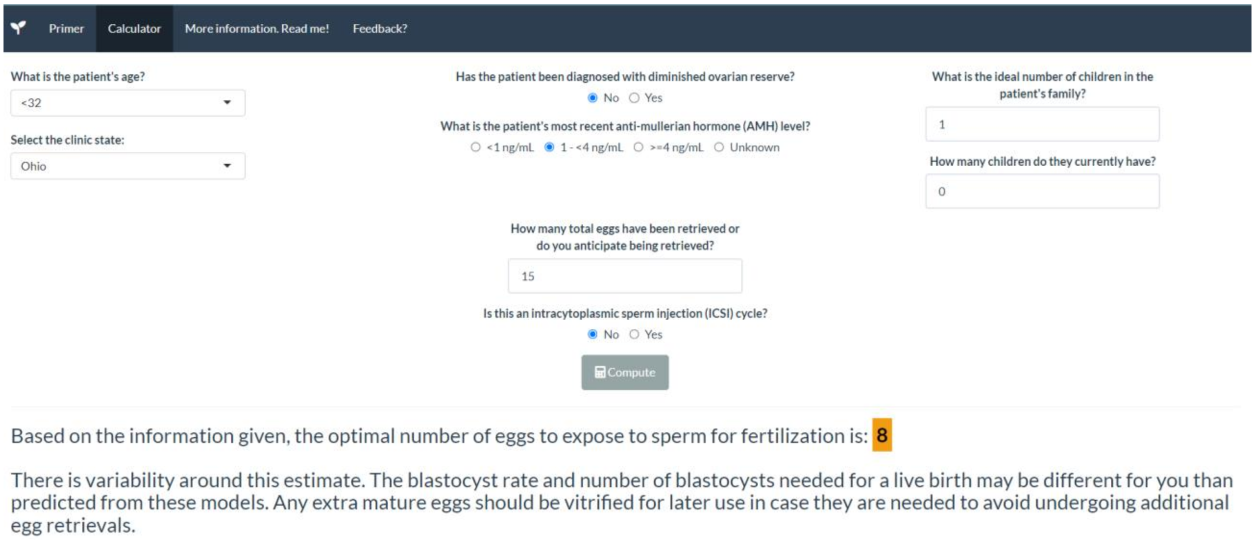

B.

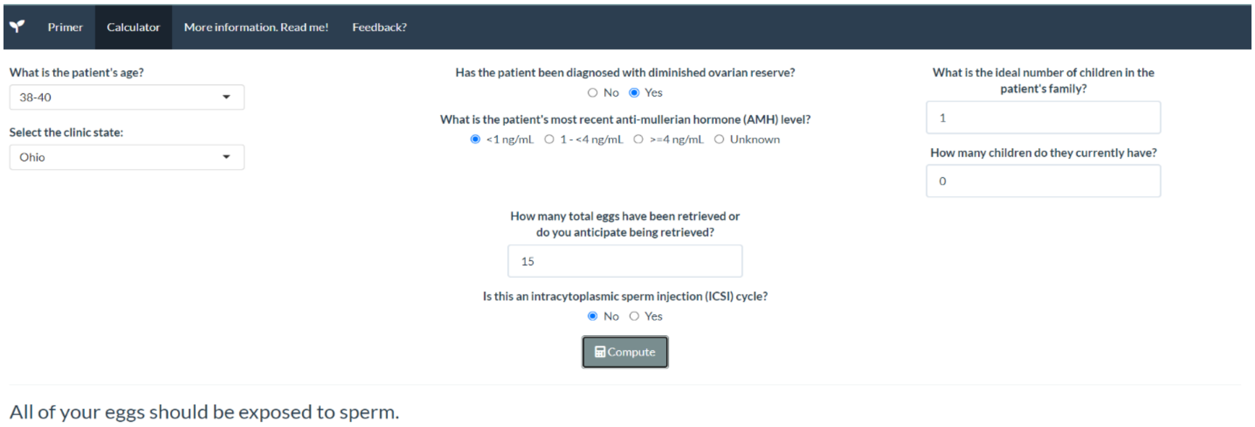

© 2023 Correia K et al. *JAMA Network Open.*
