# Supplementary material for: Development of a Model to Estimate the Optimal Number of Oocytes to Attempt to Fertilize During Assisted Reproductive Technology Treatment
Source: JAMA Netw Open. 2023 Jan 3;6(1):e2249395. doi: 10.1001/jamanetworkopen.2022.49395 (PMC9857446; doi:10.1001/jamanetworkopen.2022.49395)
Supplement: Supplement 2. — Data Sharing Statement [file jamanetwopen-e2249395-s002.pdf]

Data Sharing Statement

Correia. Development of a Model to Estimate the Optimal Number of Oocytes to Attempt to Fertilize During Assisted Reproductive Technology Treatment. *JAMA Netw Open*. Published January 03, 2023. doi:10.1001/jamanetworkopen.2022.49395

Data
Data available: No

Additional Information
Explanation for why data not available: The data used in this article cannot be shared publicly due to data protection regulations. Data are accessible to researchers associated with a SART member clinic. More information can be found here: https://www.sart.org/professionals-and-providers/research/.
